# A Case of Perinephric Pancreatic Pseudocyst Secondary to Pancreatitis

**DOI:** 10.1002/ccr3.70537

**Published:** 2025-05-28

**Authors:** Mallick Muhammad Zohaib Uddin, Wasim Ahmed Memon, Minaa Shahid, Hatem Eltaly, Saba Akram, Safna Naozer Virji, Muhammad Nadeem Ahmad, Naila Nadeem, Faheemullah Khan, Uffan Zafar

**Affiliations:** ^1^ Department of Radiology The Aga Khan University Hospital Karachi Pakistan; ^2^ Cleveland Clinic Main Campus Hospital Cleveland Ohio USA; ^3^ Department of Pathology and Laboratory Medicine The Aga Khan University Hospital Karachi Pakistan; ^4^ General Surgery Department The Aga Khan University Hospital Karachi Pakistan; ^5^ Department of Radiology Cleveland Clinic Main Campus Cleveland Ohio USA

**Keywords:** fistulous tract, pancreatic pseudocyst, pancreatitis, percutaneous drainage

## Abstract

Pancreatic pseudocyst is a rare complication of pancreatitis, characterized by an abnormal connection between the pancreas and the renal system. We present a case of a 42‐year‐old male who developed a pancreatic pseudocyst secondary to severe acute pancreatitis. The patient presented with abdominal pain, nausea, vomiting, and weight loss. Imaging studies revealed chronic pancreatitis, pseudocyst, and a fistulous tract between the pancreas and the left kidney. Management included surgical intervention, nutritional support, and medical treatment. The patient presented a month later with a post‐operative infection of the pseudocyst for which a percutaneous drainage catheter was placed under ultrasound guidance. Subsequently, he showed improvement in symptoms and laboratory parameters and was discharged in a stable condition. This case underscores the importance of a multidisciplinary approach in managing complex pancreatic pseudocysts, especially those with renal involvement, to prevent complications and to optimize outcomes.

**Summary**

- This case highlights the necessity of early recognition and adoption of a multidisciplinary approach in managing complex pancreatic pseudocysts, especially those with renal involvement. To avoid complications including infection and renal failure and to improve patient outcomes, early detection, suitable imaging, prompt surgical intervention, and close follow‐up are essential.
- The case also emphasizes the need for tailored treatment plans based on expertise and patient‐specific factors, highlighting the role of cooperation between gastroenterologists, radiologists, and surgeons in optimizing outcomes.

## 1 Introduction

Pancreatitis is associated with a range of local and systemic complications [1, 2]. However, fistula formation is noted in only a very small proportion of patients with acute pancreatitis [3]. These fistulas are generally a result of disruption of the pancreatic duct, which may be due to several etiologies such as trauma, surgical trauma, pancreatic resection, or those causing pancreatitis [4]. Pancreatic duct disruption results in fluid leakage, which causes erosion and formation of pathways, the locations of which depend on the anatomic location of the duct disruption [4]. Management of pancreatic fistulas includes medical and nutritional optimization, surgical interventions, endoscopic treatments, and catheter drainage [3, 5, 6, 7].

Pseudocysts associated with acute pancreatitis are loculated fluid collections that are rich in amylase and usually develop within 2 weeks of the onset of pancreatitis [8]. They may be intra‐ or extra‐pancreatic, are inflammatory, and lack a true epithelial lining. Just like pancreatic fistulae, pancreatic pseudocysts generally develop secondary to disruption of the pancreatic duct and resolve spontaneously unless they develop complications [8]. The management of pancreatic pseudocysts includes but is not limited to percutaneous, endoscopic, and surgical drainage, among others [9, 10, 11].

Among these complications, pancreatic pseudocyst stands as a rare but significant entity. A pancreatic pseudocyst represents an abnormal fluid collection originating from the pancreas and can arise as a consequence of acute or chronic pancreatitis and remains relatively uncommon in clinical practice.

The clinical presentation of pancreatic pseudocyst may include abdominal pain, fever, and biochemical abnormalities indicative of pancreatic injury.

In this report, we present a case of pancreatic pseudocyst formation secondary to pancreatitis in a 42‐year‐old male, highlighting the clinical presentation, diagnostic evaluation, therapeutic interventions, and outcomes. Through this case, we aim to underscore the importance of early recognition and appropriate management of pancreatic pseudocysts to prevent complications and optimize patient outcomes.

## 2 Case History/Examination

A 42‐year‐old male presented with complaints of swelling in the epigastric region for 4 months, which worsened progressively. It was associated with weight loss. His history included a single episode of acute pancreatitis 2 years ago with subsequent multiple episodes of mild epigastric pain, which were managed conservatively. On physical examination, his abdomen was soft, non‐tender, and non‐distended.

## 3 Methods (Differential Diagnosis, Investigations, and Treatment)

**FIGURE 1:**
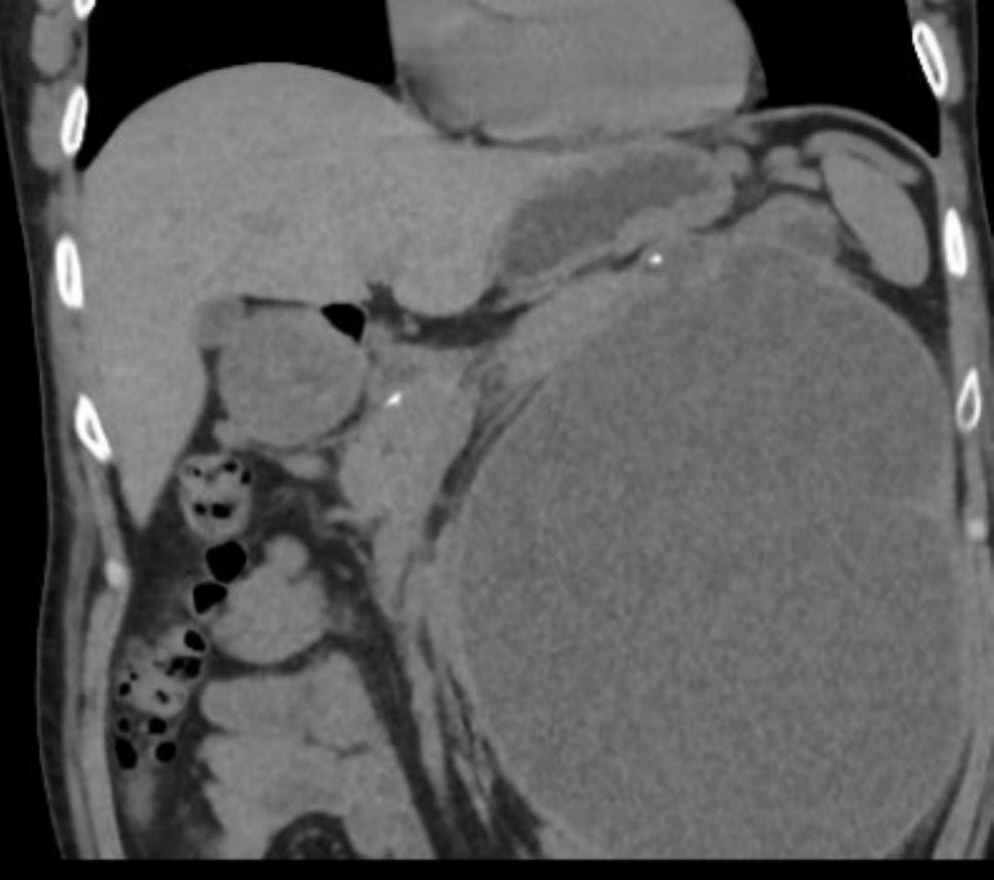
CT examination of the abdomen and pelvis shows pancreatic calcification and a large peripancreatic pseudocyst which was communicating with a dilated pancreatic duct.

**FIGURE 2:**
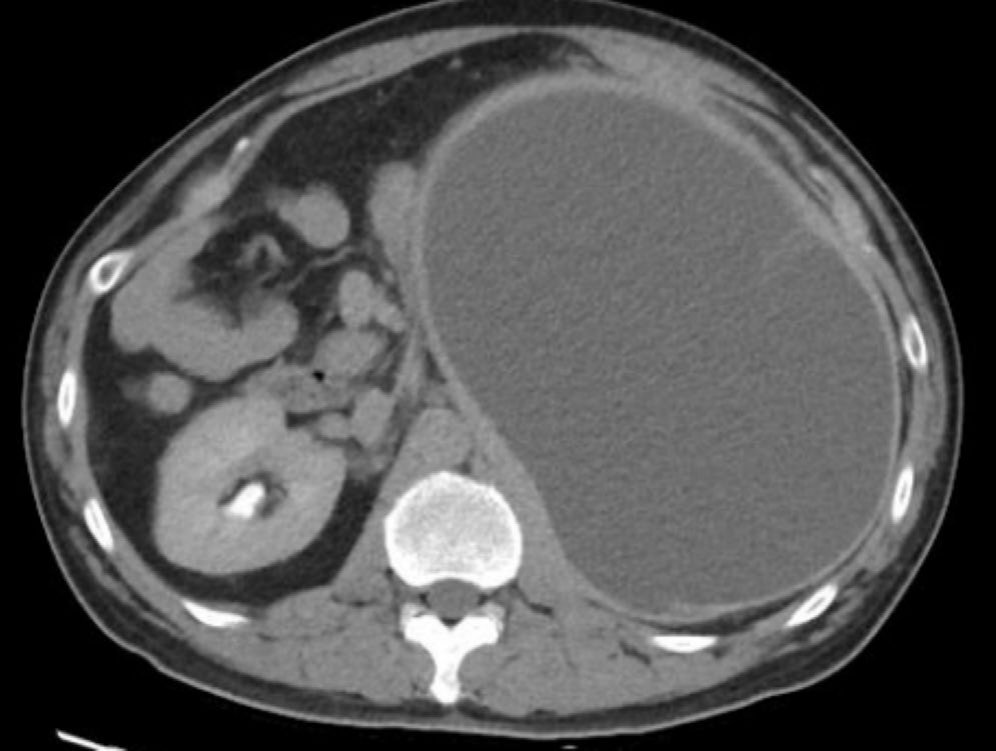
CT examination of the abdomen shows a large peripancreatic pseudocyst extending into the left perinephric region.

Contrast‐enhanced CT examination of the abdomen and pelvis showed features of chronic pancreatitis and a large perinephric pseudocyst that was communicating with a dilated pancreatic duct in the region of the pancreatic tail (Figure 1). This was causing gross compression and displacement of the left kidney posteromedially and inferiorly, with resultant splaying and significant thinning of the renal parenchyma (Figure 2) but no hydronephrosis or obstructive uropathy was observed. Laboratory investigations showed a serum creatinine level of 1.4 mg/dL (reference range: 0.6–1.2 mg/dL) which indicates mild renal impairment. Urinalysis revealed no hematuria or proteinuria.

**FIGURE 3:**
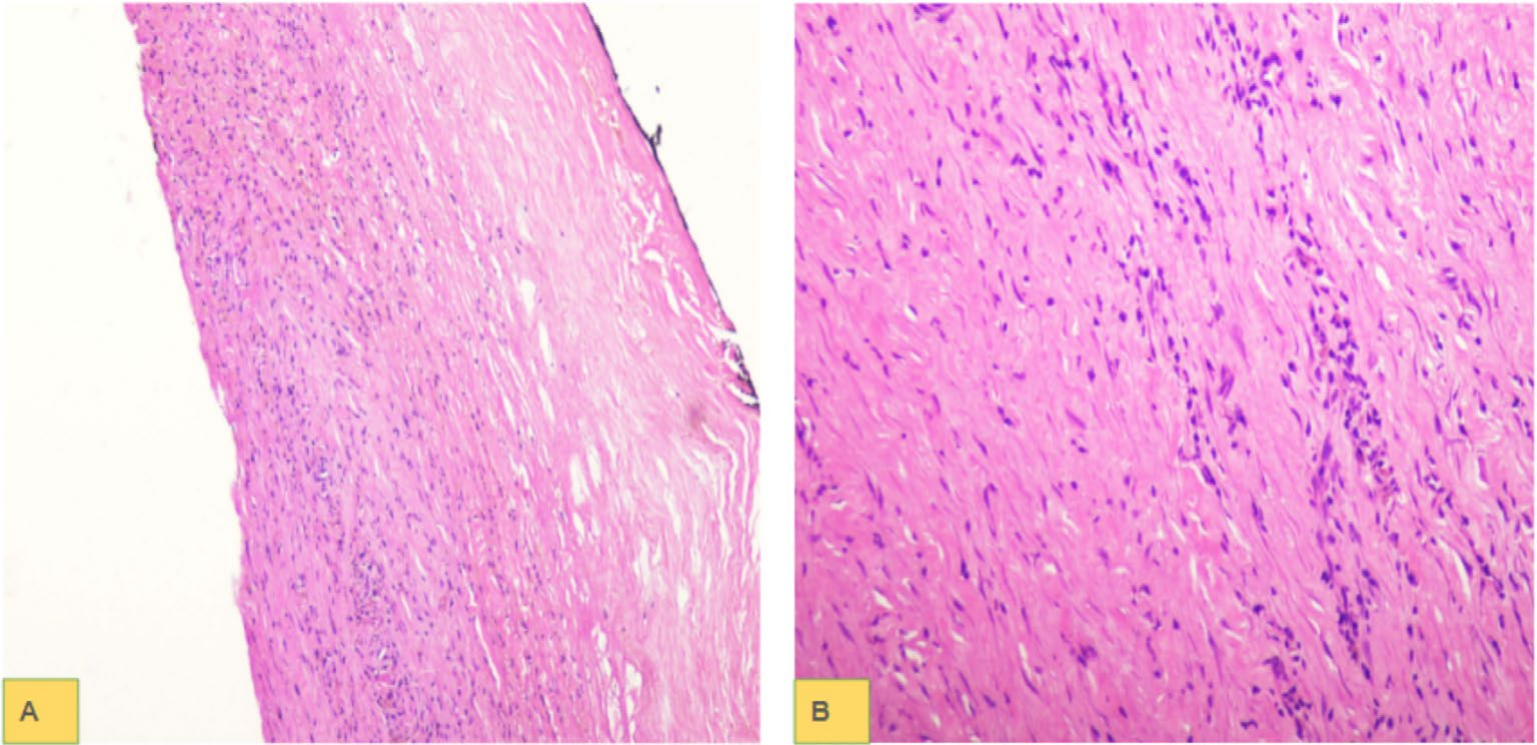
Low power (A) and high power (B) images show densely fibrosed cyst walls with lymphocytic infiltrate. No epithelial lining is seen.

Subsequently, exploratory laparotomy and cystojejunostomy were done in which more than two liters of turbid‐appearing fluid were suctioned. The wall of the cyst was sent for histopathology. Microscopic examination showed fibro‐collagenous tissue with a lymphocytic population in a densely fibrosed background. No epithelial lining was seen in the entirely submitted cyst wall, confirming the diagnosis of a pseudocyst (Figure 3).

The decision for choosing surgical intervention over EUS‐guided drainage was based on multiple factors that include: the size and location of the pseudocyst, the presence of a fistulous tract, and the need for definitive management of the renal involvement. In addition, the EUS‐guided approach requires relevant skills, which were lacking in the gastroenterology department of our hospital. The choice between transgastric, pararenal, or other approaches depends on the availability of required skills and the comfort of the radiologist [9, 10].

## 4 Conclusion and Results (Outcome and Follow‐Up)

**FIGURE 4:**
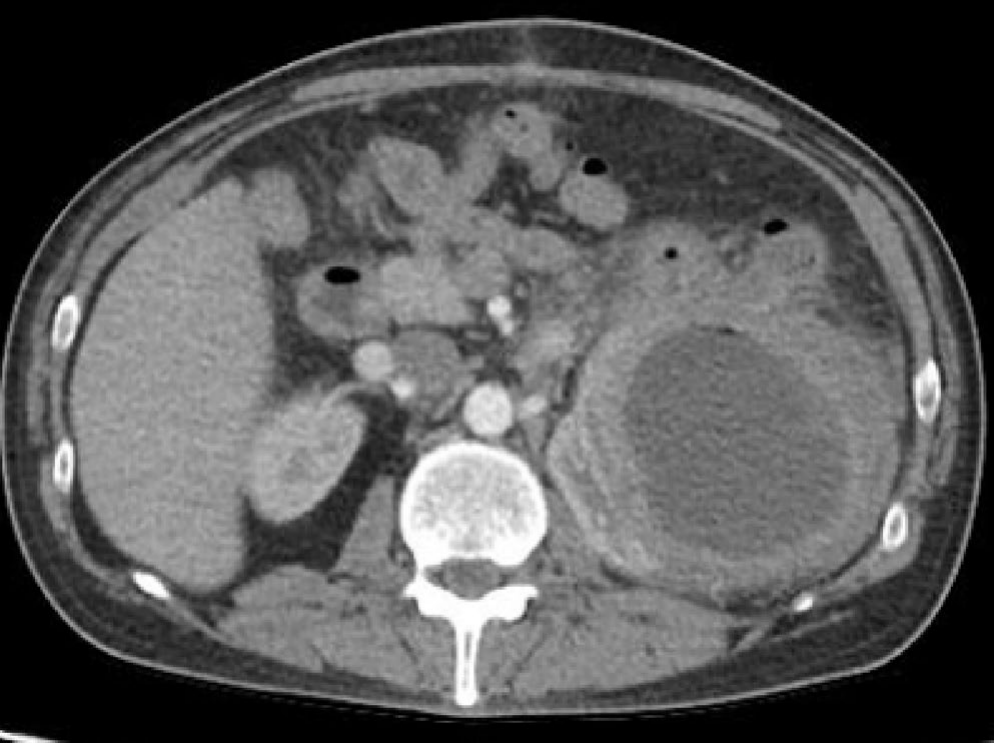
Follow‐up CT scan showed a decrease in the size of the peripancreatic pseudocyst with development enhancing thick walls along with surrounding fat stranding.

The patient presented after 1 month with a history of postoperative fever, vomiting, and fever. A follow‐up CT scan was performed, which showed an interval decrease in the size of the peripancreatic pseudocyst, with interval development of enhancing thick walls along with surrounding fat stranding suggesting infection (Figure 4).

The patient was transfused 2‐pack cell volume (PCV) due to low hemoglobin and admitted to the ward for further management. Intravenous (IV) fluids and antibiotics were continued. An ultrasound‐guided drain was placed in the pseudocyst cavity, and 120 mL of infected fluid was aspirated. Subsequently, the patient tolerated oral nutrition and was afebrile and clinically stable before being discharged. After a 3‐month follow‐up period, the patient remained asymptomatic with stable renal function (serum creatinine: 1.1 mg/dL) and no evidence of pseudocyst recurrence on imaging.

Pancreatico‐renal fistula is a rare but potentially serious complication of pancreatitis. Early recognition and appropriate management are crucial to prevent complications and improve outcomes. This case highlights the importance of a multidisciplinary approach involving gastroenterologists, radiologists, and surgeons in the management of pancreatic pseudocysts. Further research is needed to better understand the pathophysiology and optimal management strategies for this uncommon condition.

## 5 Discussion

Pancreatic pseudocysts are organized loculated simple fluid collections that lack an epithelial lining, contain a fibro‐inflammatory lining made up of fibrous and granulation tissue, and persist for more than 4 weeks after the onset of pancreatitis. They are usually in the peripancreatic location and less commonly in other locations.

Renal subcapsular extension of a pancreatic pseudocyst is extremely rare and may mimic a renal cortical cyst. Perirenal pseudocysts may compress, displace, distort, or depress the kidney. These may result in “page kidney” which refers to renin‐angiotensin‐mediated hypertension secondary to renal hypoperfusion following long‐standing compression of renal parenchyma by a subcapsular collection [12, 13, 14]. Early management of perirenal pseudocysts is, therefore, of high significance.

Pancreatico‐renal fistula is a rare complication of pancreatitis. Its pathogenesis typically involves the disruption of pancreatic duct integrity due to inflammation, ductal obstruction, or tissue necrosis [4]. In the background of pancreatitis, inflammation can lead to the formation of abscesses or pseudocysts [8], which may erode into adjacent structures with resultant fistula formation [3].

The clinical presentation depends on the extent of the fistula and associated complications. Patients typically present with a constellation of symptoms including abdominal pain, urinary symptoms, electrolyte abnormalities, or recurrent urinary tract infections. These may be associated with raised serum amylase and lipase levels. Diagnosis may be confirmed through imaging studies such as CT, magnetic resonance imaging (MRI), or endoscopic retrograde cholangiopancreatography (ERCP). In case renal involvement is suspected, renal scintigraphy may provide additional functional information, particularly in assessing renal perfusion and detecting complications such as page kidney. Although renal scintigraphy was not performed in this case, its utility in similar cases should be considered for future reference [12, 13].

Managing pancreatic pseudocysts requires a multidisciplinary approach tailored to the individual patient's clinical presentation and underlying etiology [9, 10, 11]. Conservative measures such as fluid resuscitation, pain control, and antibiotics may be initiated to stabilize the patient and control symptoms. In cases of persistent symptoms or complications such as infection or renal dysfunction, more invasive interventions may be warranted. Endoscopic therapy, including ERCP with stent placement or sphincterotomy, can facilitate drainage of the pancreatic duct and promote closure of the fistulous tract. Surgical intervention may be necessary in cases of failed endoscopic therapy, extensive pancreatic necrosis, or recurrent complications. Surgical options include fistula excision, pancreatic duct ligation, or partial pancreatectomy, depending on the extent of pancreatic involvement and the patient's overall condition. In our case, surgical internal drainage was performed on first admission. However, the patient was again admitted with an infected pseudocyst, and an ultrasound‐guided pigtail drainage catheter was placed.

The prognosis of pancreatic pseudocysts depends on the severity of pancreatic inflammation, the extent of adjacent tissue involvement, and the timely initiation of appropriate treatment. With prompt diagnosis and early intervention, most patients experience resolution of symptoms and improvement in pancreatic and renal function. However, complications such as recurrent pancreatitis, infection, or renal insufficiency may occur, particularly in cases of delayed diagnosis or inadequate treatment. Long‐term follow‐up is essential to monitor the recurrence of symptoms and ensure optimal outcomes for patients with pancreatic pseudocysts.

## Author Contributions

**Mallick Muhammad Zohaib Uddin:** conceptualization, data curation, investigation, project administration, writing – original draft, writing – review and editing. **Wasim Ahmed Memon:** writing – original draft, writing – review and editing. **Minaa Shahid:** conceptualization, data curation, supervision, validation. **Hatem Eltaly:** conceptualization, data curation, investigation, supervision, validation, writing – original draft. **Saba Akram:** conceptualization, data curation. **Safna Naozer Virji:** conceptualization, data curation. **Muhammad Nadeem Ahmad:** supervision, validation. **Naila Nadeem:** supervision, validation. **Faheemullah Khan:** conceptualization, supervision, validation. **Uffan Zafar:** conceptualization, data curation, investigation, project administration, writing – original draft, writing – review and editing.

## Ethics Statement

The authors have nothing to report.

## Consent

Written informed consent was obtained from the Participant.

## Conflicts of Interest

The authors declare no conflicts of interest.

## Data Availability

The data supporting the findings of this study are available from the corresponding author upon reasonable request.
